# Outcomes of pelvic radiotherapy with boost strategies in high nodal-risk prostate cancer: A phase 2 prospective trial

**DOI:** 10.1016/j.ctro.2026.101175

**Published:** 2026-04-23

**Authors:** Camilla Thellenberg Karlsson, Kristina Notstam, Björn Tavelin, Kristina Lundqvist, Per Fransson, Karin Söderkvist

**Affiliations:** a Department of Diagnostics and Intervention, Oncology, Umeå University, 901 87 Umeå, Sweden; b Department of Chemistry, Umeå University, 901 87 Umeå, Sweden; c Department of Nursing, Umeå University, 901 87 Umeå, Sweden

## Abstract

**Purpose/Objective:**

The optimal radiotherapy strategy for prostate cancer (PC) patients with high nodal risk remains debated. This prospective phase II study reports long‑term clinical outcomes, toxicity, and patient‑reported outcomes in men with PC treated with whole‑pelvis radiotherapy and dose escalation to MRI‑identified intraprostatic lesions and PET‑positive pelvic lymph nodes.

**Materials/Methods:**

Eighty‑five PC patients with high nodal risk or up to three pelvic nodal metastases were enrolled between 2013 and 2017. Radiotherapy delivered 77 Gy to the prostate and 56 Gy to pelvic nodes in 35 fractions, with escalation to 70 Gy for PET‑positive nodes and 84 Gy for MRI‑defined intraprostatic lesions when feasible. Endpoints included biochemical progression‑free survival, overall survival, toxicity, and longitudinal patient‑reported outcomes.

**Results:**

Seventy-eight patients underwent radiotherapy. Of these, 42 received an intraprostatic boost and were classified as the per-protocol population. Median follow‑up was 7.8 years, and 26% had N1 disease. For the full cohort, five‑year biochemical progression‑free survival was 76%, with poorer results among patients with RECIST‑positive nodal involvement. Five‑year overall survival was 95%. Acute grade ≥ 2 genitourinary and gastrointestinal toxicities occurred in 33 and 23%, respectively, and decreased over time. No grade ≥ 3 gastrointestinal toxicity was observed. Patient‑reported outcomes showed low long‑term urinary and bowel bother.

**Conclusion:**

Whole‑pelvis radiotherapy with targeted dose escalation was well tolerated. The high proportion of patients not receiving an intraprostatic boost underscores methodological challenges and emphasises the need for standardised imaging interpretation and delineation guidelines.

## Highlights

• **Study Overview:** Single-arm trial of pelvic RT + intraprostatic boost for high/very high-risk prostate cancer (inc. N1).
• **Efficacy:** 5-year biochemical progression-free survival was 76% overall and 80% for patients without nodal disease.
• **Safety:** Favorable profile; no grade 3–4 GI toxicity and low rates ≥ 2 GU toxicity despite using targeted focal boosts.
• **Patient Outcomes:** Low 5-year bother scores, though urinary symptoms reflect intensive treatment and high baseline burden.
• **Conclusions**: Pelvic RT with dose escalation was well tolerated; results show a need for better boost imaging standards.

## 1 Introduction

The risk of pelvic lymph node involvement increases with higher tumour stage, PSA levels and tumour grade. However, the use of whole-pelvic nodal radiotherapy (WPRT) has been controversial over the past decades. Early randomized trials were limited by the inclusion of low-risk patients, insufficient treatment doses to the prostate and inadequate pelvic field-size [1], [2]. More recently, the randomized POP-RT trial demonstrated improved biochemical failure-free survival (bFFS) and disease-free survival (DFS) with prophylactic WPRT compared to prostate-only RT (PORT) in high-risk patients without nodal metastases, although no difference in overall survival (OS) was observed [3]. Notably, the POP-RT protocol did not incorporate focal intraprostatic boosting.

Dose escalation to the prostate in high-risk prostate cancer (PC) has demonstrated a higher proportion of patients without relapse after definitive radiotherapy (RT), albeit at the cost of increased toxicity [4], [5], [6], [7]. Local recurrence after RT usually occurs at the same site as the dominant intra-prostatic lesion (DIL) at baseline [8], [9], [10]. Thus, a focal boost to an image based DIL is proposed as an *iso*-toxic method to improve treatment outcome [11], [12], [13], [14]. Randomized data from the FLAME trial demonstrated that focal boosting up to 95 Gy improved biochemical disease-free survival (bDFS) in patients with intermediate and high-risk PC, without significant increase in toxicity [15], [16]. Despite the level 1 evidence from the FLAME trial, focal boosting has not yet been widely adopted, largely due to methodological challenges in implementation.

[1–3]This phase II trial was conducted to evaluate the outcomes of WPRT incorporating dose escalation to the MRI-defined dominant intraprostatic lesion and PET-positive lymph nodes in a PC population with high predicted nodal risk or up to three pelvic nodal metastases.

## 2 Materials and Methods

### 2.1 Study design and participants

The study was designed as a single centre phase II trial including protate cancer patients with a high-risk of lymph node metastasis accepted for definitive RT between 2013–2017. Key eligibility criteria were an estimated risk of lymph node involvement > 15% according to the MSKCC Prostate Cancer Nomogram (www.nomograms.org). Exclusion criteria were distant metastases (M1), > 3 pelvic lymph node metastases, previous pelvic irradiation, TURP within six months, IPSS score > 19 and contraindications for MRI. Additionally, all patients underwent a parallel exploratory imaging protocol involving serial MRI and PET scans during treatment.

The full study protocol is provided in the Supplementary Protocol.

The trial was conducted in accordance with Good Clinical Practice guidelines and the Declaration of Helsinki and has ethical approval (Dnr 2013/154–31 and 2015/75–32). It has been registered at ClinicalTrials.gov (NCT01962324; study acronym: PARAPLY-1 (**PA**tients receiving **RA**diotherapy for **P**rostatic cancer, with high risk for **LY**mph node metastasis).

### 2.2 Procedures and radiotherapy

All patients were staged by bone scintigraphy and ^18F^Acetate-PET/CT (ACE-PET). At inclusion, before the start of androgen deprivation therapy (ADT) and fiducial marker implantation, a multiparametric (mp) MRI was performed. This included T1-, T2, diffusion-weighted (DW) and contrast enhanced (DCE) MRI. After 14 patients had been included, a PET/MRI was installed in the department and thereafter patients performed an ACE-PET/mpMRI. Since ACE-PET is no longer the method of choice for staging, an additional post hoc staging according to RECIST1.1 by a radiologist was performed for the analyses. All patients received neoadjuvant androgen deprivation therapy (ADT) 3 months before RT and 3 months concomitant with RT followed by 6 months of adjuvant antiandrogen treatment with bicalutamide. Before the start of RT, three gold fiducial markers were implanted in the prostate and a CT with a slice thickness of 2 mm and a second mpMRI of the prostate were performed for treatment planning. To minimize bowel movements all patients were injected with 1 mg Glucagon s.c. prior to MRI. Scanning was performed on a flat tabletop using the same set-up as during RT.

The CTV included the entire prostate (CTVT), vesicula seminalis (CTVT2) and the pelvic lymph nodes (CTVN). CTVN was outlined according to RTOG guidelines from 2009, with the exception of presacral lymph nodes. A 6 mm isotrop margin was added for the prostate PTV (PTVT_77), and 7 mm for the vesicula seminalis (PTVT2_56) /pelvic lymph nodes (PTVN_56). The intraprostatic gross tumour volume (GTVT) used for focal boosting was outlined in mpMRI with support of the diagnostic ACE-PET/CT or ACE-PET/mpMRI and the histological report. An isotrop margin of 2 mm was added for the PTVT_84 and cropped so that no part of PTVT_84 extended into organs at risk (OARs) such as urethra, bladder, or rectum/anal canal. Lymph nodes suspected of macroscopic tumour growth in the staging PET-CT were outlined as GTVN with an isotrop margin of 7 mm to generate PTVN_70. The delineation of the GTVT and GTVN, if applicable, could be opted out by the attending physician if no intraprostatic lesion or lymph nodes could be visualized in the planning mpMRI/CT. The definition of volumes followed the recommendations made by ICRU in Report 50, 62 and 83 for photon beam therapy [17], [18], [19].

The treatment was delivered with a simultaneous integrated volumetric arc technique at the same time of day, 5 days/week. The prescribed doses were 77 Gy in 35 daily fractions (EQD2 80 Gy (α/β 3)) to the PTVT and 56 Gy/35 fr (EQD2 50 Gy) to PTVT2/ PTVN. The intraprostatic focal boost was prescribed a total dose of 84 Gy/35 fr (EQD2 90 Gy) and positive pelvic lymph nodes received 70 Gy/35 fr, if applicable.

The patients were instructed for bladder filling and prescribed a bowel-regulating agent. A daily online position verification protocol with the implanted fiducial markers was used. A cone beam CT (CBCT) was performed weekly to assess the relation between the vascular/skeletal structures and the prostate. If a patient transfer exceeded 5 mm from set up (skeletal structure) to match the fiducials, a CBCT was performed, and assessed by the treating physician if additional action was needed **(**Supplementary Protocol**).**

### 2.3 Follow-up and assessment of side effects

Side effects were graded by the treating physician according to the Radiation Therapy Oncology Group (RTOG) acute and late grading scales before start of RT, at the end of RT and at 6, 12, 18, 24, 30 and 36 months after the end of RT. All patients received the validated patient-reported outcome (PRO)-questionnaire Prostate Cancer Symptom Scale (PCSS) [20], [21], [22] before the start of RT, at the end of RT, at 6 months, and at 1, 2, 3 and 5 years. PCSS focuses on urinary, bowel and sexual function and scored 0–10, where a higher value refers to more symptoms/worse function. Minimally clinical important changes in PRO was defined as in the Hypo-RT-PC trial [29]) and based on the method by Osoba [23] All patients received ADT, and we did not focus on the sexual function in the evaluation.

### 2.4 Study outcomes

The primary endpoint was biochemical progression free survival (bPFS), defined as the time from inclusion to the first occurrence of PSA progression according to the *Phoenix* definition (nadir PSA + 2 ng/mL).

Secondary efficacy endpoints included DFS, defined as the time from inclusion to either biochemical recurrence or radiological progression (whichever occurred first), and overall survival (OS), defined as the time from inclusion to death from any cause.

Secondary safety and patient reported endpoints comprised physician-scored genitourinary (GU) and gastrointestinal (GI) side effects, assessed according to the RTOG criteria, as well as PRO-questionnaires with PCSS.

### 2.5 Statistical analysis

Survival analysis, i.e. bPFS and OS, was performed in R (v4.3.2; R Core Team 2023) and the package survival. The analysis was performed with endpoints specified above, and with the censoring time as the date of last follow-up. Significant difference in survival curves was tested using a logrank test. To calculate median follow-up time, reversed Kaplan-Meier was performed in the R-package prodlim.

Differences between two timepoints (baseline and 5-year follow-up) were tested with a paired-samples *t*-test. The test was performed with SPSS 28.0 (SPSS Inc., Chicago, IL, USA). All tests were performed with a significance level 5%.

All patients that signed the informed consent and were treated according to the protocol with WPRT and a simultaneously integrated boost to the prostate were considered the intention to treat population (ITT). Patients that received the additional protocol-stipulated intraprostatic boost were considered the per protocol population (PP). All survival analyses were performed for both populations.

## 3 Results

**Fig. 1:**
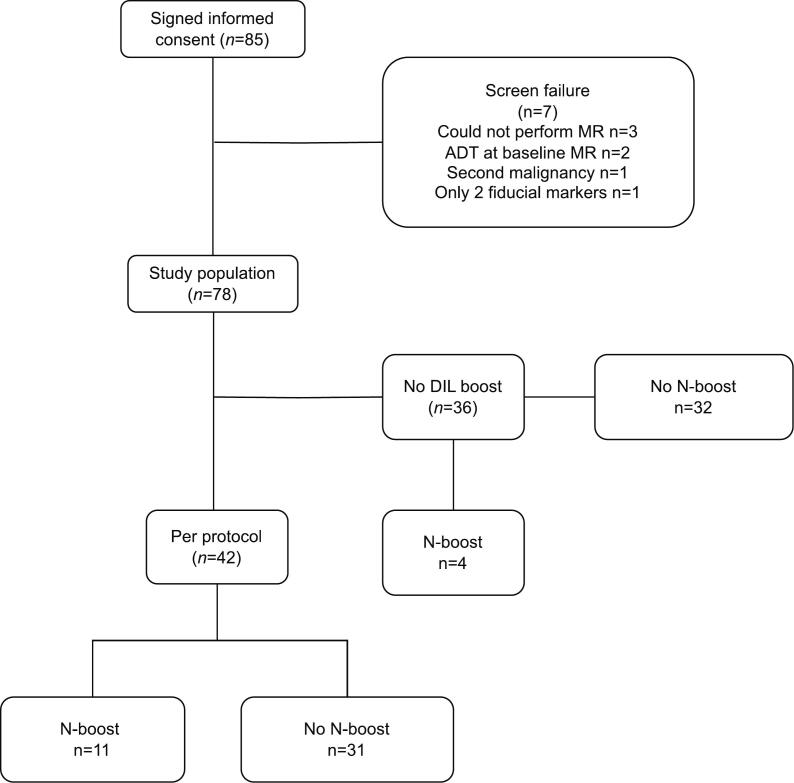
**CONSORT flow diagram.** CONSORT diagram of patients included in the trial who received treatment per protocol, along with the distribution of boost to DIL and/or lymph nodes. **Abbreviations:** DIL − Dominant intraprostatic lesion.

A total of 85 consecutive patients were enrolled in the study between 2013 and 2017. Seven patients were excluded before treatment due to screening failures (inability to perform MRI, baseline ADT, synchronous malignancy, or inadequate fiducial marker placement). The remaining 78 patients constituted the intention to treat (ITT) population. The DIL could not be identified in 36 patients, and they were therefore not considered eligible for intraprostatic boosting. 42 patients had an identifiable DIL and received an intraprostatic boost, they are here after referred to as the per protocol (PP) population. A consort diagram of the study is displayed in Fig. 1.

Baseline characteristics for the full intention‑to‑treat cohort showed a median age of 71 years and a median follow‑up of 7.8 years. Post-hoc analyses demonstrated that all except four patients fulfilled the EAU/NCCN high-risk criteria (cT3 or PSA > 20 or Gleason 8–10). The remaining four patients did not meet the high-risk definition and would instead have been classified as having unfavourable intermediate-risk disease. Furthermore, 34 patients –(44%) would have been eligible for abiraterone according to the STAMPEDE criteria (two or more of: Gleason score ≥ 8, PSA ≥ 40, ≥cT3 and/or N1) [24].

Twenty patients (26%) had N1 disease on ACE-PET or RECIST assessment, and five patients (6%) presented with PSA levels above 100 ng/mL. The median estimated risk of pelvic nodal involvement was 36% (Roach) and 38% (MSKCC), excluding patients with radiologically visible lymph nodes (Table 1). Of the 20 patients with N1 disease, 5 did not receive a nodal boost. In these cases, the involved lymph node was deemed unsuitable for image guided dose escalation, either due to difficulties visualising the node on CBCT or because boosting would have compromised organs at risk, most commonly small bowel constraints.

**Table 1:** Baseline characteristics for the overall cohort and for patients with and without intraprostatic boost.

**Characteristics**	**All patients (ITT) (n = 78)**	**Intraprostatic boost (PP)** **(n = 42)**	**No intraprostatic boost (n = 36)**
**Age (years)**			
Mean (range)	70 (56–82)	71 (59–82)	69 (56–78)
**PSA (ng/ml)**			
Median (range)	20 (2–281)	17 (2–119)	25 (5–281)
**Gleason score n (%)**			
<7	6 (8)	3 (7)	3 (8)
7	19 (24)	8 (19)	11 (31)
≥8	53 (68)	31 (74)	22 (61)
**T stage n (%)**			
T1c	11 (14)	5 (12)	6 (17)
T2	42 (54)	23 (55)	19 (53)
T3	25 (32)	14 (33)	11 (31)
**N stage n (%)**			
N0	58 (74)	30 (71)	28 (78)
N1 (all)	20 (26)	12 (29)	8 (22)
• N1 (RECIST)	6 (8)	3 (7)	3 (8)
• N1 (Ac-PET)	19 (24)	12 (29)	7 (19)
Nodal risk % (MSKCC)*			
Median	38	43	36
Nodal risk % (Roach)*			
Median **NCCN/EAU risk group (%)** Unfav. intermediate High-risk STAMPEDE eligibility (%)**	36 4 (5) 74 (95) 34 (44)	36 2 (5) 40 (95) 19 (45)	36 2 (6) 34 (94) 15 (42)

*Only for the node negative patients (n = 58).

**Eligibility defined according to STAMPEDE (≥2 of: Gleason ≥ 8, PSA ≥ 40, ≥cT3 and/or N1).

**Fig. 2:**
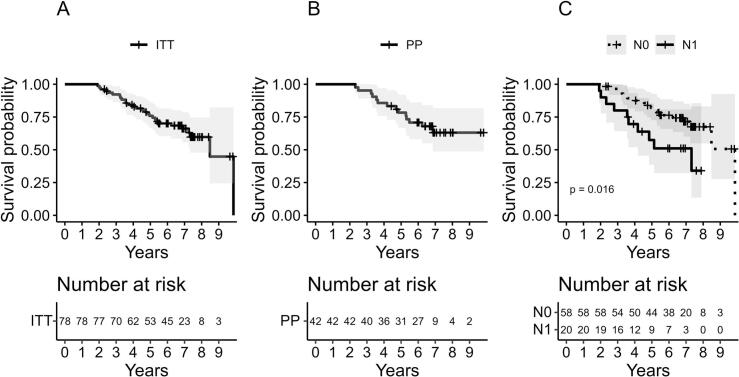
**Bpfs in the itt, pp and nodal subgroups.** Kaplan-Meier curves showing bPFS for the overall cohort, ITT (A) PP (B) and comparison of bPFS between patients with N0 disease (dashed line) and N1 disease (solid line) (C). **Abbreviations:** bPFS = Biochemical progression‑free survival; N = TNM classification of malignant tumours (UICC).

For all patients (ITT), the 5-year biochemical progression free survival (bPFS) was 75.9%. Nodal involvement was strongly associated with inferior outcomes: patients with N1 disease had a 5-year bPFS of 57.5% compared with 82.0% for N0 patients (p = 0.016) **(**Fig. 2**)**. Among N1 patients, those classified as having measurable nodal disease according to RECIST 1.1 had particularly poor outcomes, with a 5-year bPFS of 25% (n = 6). Sensitivity analysis excluding EAU/ NCCN intermediate patients did not alter the bPFS, it remains 75.9% at five years.

No patients progressed without rising PSA, therefore we have not calculated disease free survival separately. The 5-year overall survival for all patients (ITT) was 94.9%**.** During follow-up, 12 deaths occurred, two attributable to prostate cancer and ten to other causes.

**Fig. 3:**
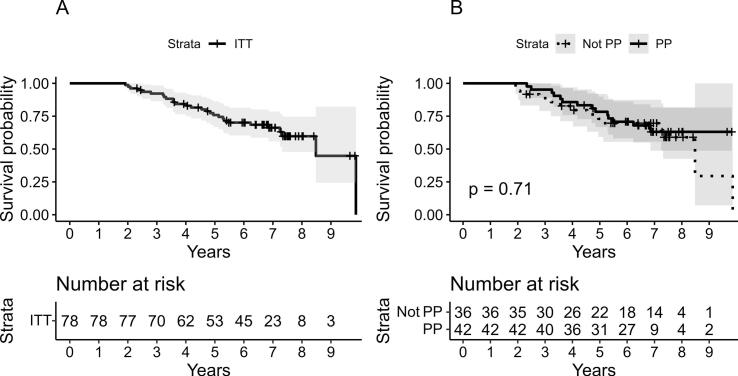
**Bpfs in the overall cohort and pp subgroups.** Kaplan-Meier curves showing bPFS for the overall cohort (A) and comparison of bPFS between patients treated per protocol (PP) and those included in ITT but not treated per protocol (Not‑PP) (B). **Abbreviations:** bPFS = Biochemical progression‑free survival; ITT = Intention‑to‑treat; PP = Per protocol.

Among patients that received an intraprostatic boost (PP), the corresponding 5-year bPFS was 78.3%, compared with 73.0% among those treated without a DIL boost (p = 0.71) **(**Fig. 3**).** The mean dose to the DIL (GTV_DMean) among the 31 patients with available dosimetric data was 83.2  Gy (range 81.5–84.7  Gy; 95% CI 82.9–83.5  Gy). Detailed dose-volume data are provided in Supplementary **(Table S1).**

### 3.1 Toxicity (RTOG)

In the overall study population, acute grade ≥ 2 GU was observed in 33.3% of patients at the end of RT and 11.6% at the 6-month FU. Five patients (6.4%) experienced acute grade 3 GU toxicity at the end of treatment, and two patients (2.6%) had grade 3 toxicity at 6 months. At the final FU (36 months), the incidence of grade ≥ 2 GU toxicity was 11.6%, with no grade ≥ 3 toxicity recorded **(**Fig. 4A**).**

**Fig. 4A:**
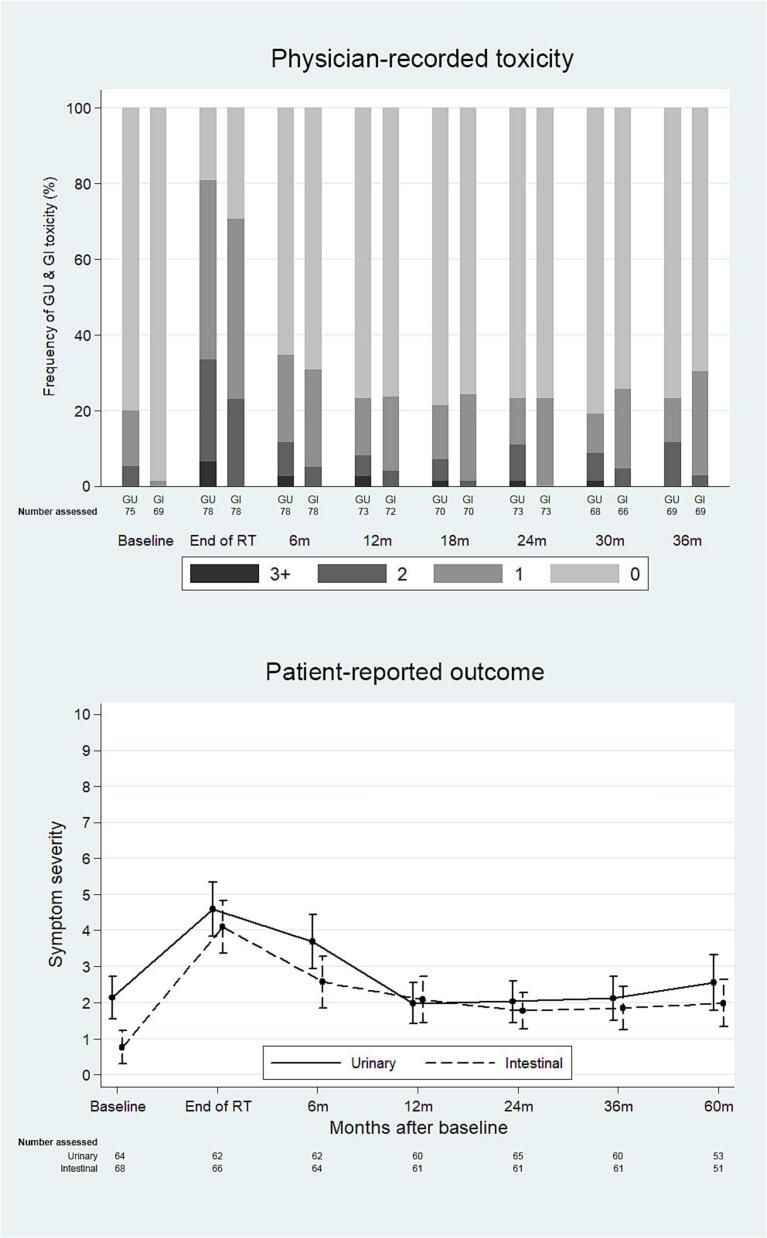
**GU and GI toxicity and urinary/bowel bother for all patients, ITT.** Physician-recorded GU and GI toxicity (RTOG, scale 0–5) and patient-reported overall urinary and bowel bother mean score (PCSS, scale 0–10). **Abbreviations**: RTOG − Radiation Therapy Oncology Group, PCSS − Prostate Cancer Symptom Scale.

Acute GI toxicity of grade ≥ 2 occurred in 23.1% of all patients at the end of treatment, decreasing to 5.1% at 6 months. No grade 3 or 4 GI toxicity was recorded at any assessment. At 36 months, two patients (2.9%) were reported with grade 2 GI toxicity **(**Fig. 4A**).** No differences in GU or GI toxicity were observed between patients treated with an intraprostatic boost (PP) and those treated without a DIL boost **(**Fig. 4B**, Table S2)**.

**Fig. 4B:**
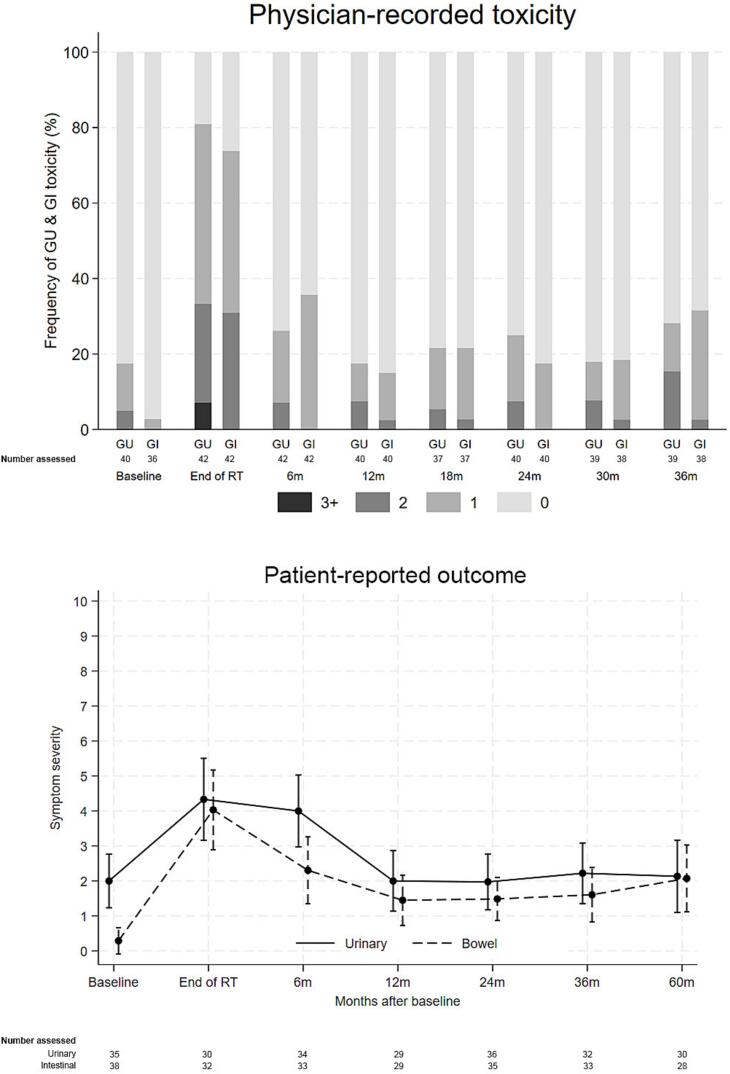
**GU and GI toxicity and urinary/bowel bother for patients with intraprostatic boost (PP).** Physician-recorded GU and GI toxicity (RTOG, scale 0–5) and patient-reported overall urinary and bowel bother mean score (PCSS, scale 0–10). **Abbreviations**: RTOG − Radiation Therapy Oncology Group, PCSS − Prostate Cancer Symptom Scale.

### 3.2 Pros (PCSS)

A total of 444 PRO-questionnaires were evaluated. At the 5-year FU, 54 of 74 eligible patients (73%) returned the questionnaire.

For the entire population, mean overall urinary bother scores (scale 0–10) were 2.1 (95% CI 1.6–––2.7) before start of RT, 4.6 (95% CI 3.8–5.3) at the end of RT, 2.0 (95% CI 1.4–––2.6) at 1 year and 2.6 (95% CI 1.8–––3.3) at 5 years, Fig. 4A. The proportion of patients reporting moderate or severe overall urinary problems (≥ 5 on the 0–10 scale) was 19% before start of RT, 53% at end of RT and 23% at 5 years.

Mean overall bowel bother scores was 0.8 (95% CI 0.3–––1.2) before start of RT, 4.1 (95% CI 3.4–––4.8) at the end of RT and 2.1 (95% CI 1.4–––2.7) at the 1-year FU, and 2.0 (95% CI 1.3–––2.6) at 5 years (Fig. 4A). The proportion of patients reporting moderate or severe overall bowel problems was 7% before RT, 41% at end of RT and 22% at 5 years. Across all follow-up time points, no significant differences in urinary or intestinal bother were observed between patients treated with and without an intraprostatic boost **(**Fig. 4B**, Table S2)**.

At the 5-year FU, 21 of 47 patients (45%) experienced a clinically relevant deterioration in overall urinary problems (defined as ≥ 1 over baseline). For bowel bother, 23 of 49 patients (47%) showed a clinically relevant deterioration at 5 years **(Table S3)**.

## 4 Discussion

In this prospective phase II trial evaluating WPRT with intraprostatic and nodal dose escalation, we observed a 5-year bPFS of 76% in the ITT population. When excluding patients with RECIST-defined nodal involvement, the bPFS increased to 80%. These results reflect the very high-risk characteristics of the cohort and the poor prognosis of patients with measurable cN1 disease, in whom bPFS was approximately 25%. Post-hoc re-stratification using contemporary EAU and NCCN classifications (see Table 1) confirms that the cohort represents a genuinely high-risk population by 2026 standards, with only four patients potentially classifiable as unfavourable intermediate risk. These patients did not differ in bPFS from the remainder in a sensitivity analysis. The findings thus remain relevant to contemporary practice, particularly in settings where PSMA PET is not yet universally available.

The outcomes should be interpreted in the context of existing evidence from trials investigating focal boosts and pelvic irradiation. In the FLAME trial, focal intraprostatic boosting improved 5-–year bDFS from 85% to 92% [15]. However, the FLAME population differed from ours, with 15% intermediate risk patients, no N1 disease, and higher prescribed doses to the dominant intraprostatic lesion. Such differences in disease burden, staging accuracy, and boost dose likely account for the considerably higher– efficacy reported in FLAME. Similarly, the POP-RT trial demonstrated– 92% 5–year bPFS in its WPRT arm [3], but N1 patients were specifically excluded and the vast majority underwent PSMA-PET–-based staging. By contrast, the PARAPLY cohort included a substantial number of patients with PET–-positive nodal disease, staged using acetate-–PET, a modality with lower sensitivity for detecting both nodal and distant metastases [25]. The elective field in POP-RT also extended cranially to the common iliac lymph nodes, whereas the present study limited coverage to the promontory. These differences in nodal staging and treatment volumes help contextualise the higher failure rates observed– in PARAPLY.

The DELINEATE trial provides another relevant comparator. In cohort C, patients received 74  Gy to the prostate with an 82  Gy intraprostatic boost, together with WPRT to 60  Gy in 37 fractions [26], resulting in more favourable bPFS outcomes than those observed in our study. Notably, more than 80% of patients in cohort C received long-–term ADT. Another important difference between the protocols is that DELINEATE included the seminal vesicles in the high-dose CTV, whereas Swedish practice at the time of PARAPLY generally limited– the high-dose– CTV to the prostate and proximal seminal vesicles. This distinction may partly explain the observed outcome differences.

Despite these differences, toxicity outcomes in the present study were favourable and consistent with other WPRT trials. No grade ≥ 3 GI side effects were observed, and side effects from GU was within the range reported in POP-–RT and PIVOTAL [27], [28], supporting the tolerability of WPRT delivered with modern IMRT techniques.

Patient-reported outcomes were collected up to five years after radiotherapy, with a response rate of 73%. Overall bother levels remained low, although a clinical deterioration in urinary symptoms was observed over time. Compared with the HYPO–-RT–-–PC trial— which used the same questionnaire but included predominantly intermediate-risk– patients (89%) [29]- patients in the PARAPLY-1 cohort reported slightly higher urinary bother already at baseline, and this difference persisted throughout long-term follow–-up–. This pattern may reflect both the more intensive treatment regimen used in PARAPLY-1 and the higher tumour stage among participants. PRO findings from FLAME, PIVOTAL, and DELINEATE appear broadly comparable, though direct comparisons are limited by differences in assessment instruments [15], [26], [28].

An important factor to consider when interpreting our findings is the systemic therapy used. The protocol-–mandated regimen − 6 months of LHRH agonist followed by 6 months of bicalutamide − constitutes undertreatment by contemporary standards. Current EAU and NCCN guidelines recommend ≥ 12 months of LHRH-based– ADT, and based on modern STAMPEDE criteria, a large proportion of the PARAPLY-1 cohort would today be candidates for intensified therapy with abiraterone [24]. The absence of such intensification likely contributed to inferior systemic control, especially in cN1 patients.

Strengths of the study include its prospective design, systematic administration of WPRT to all participants, long follow-up, comprehensive toxicity reporting, and collection of longitudinal PRO data.

Several limitations warrant careful consideration. First, although the trial incorporated an intraprostatic boost, technical restrictions − primarily limited visualisation of the DIL on MRI- reduced adherence to the protocol-specified intraprostatic dose escalation. Moreover, the study was performed prior to the standardized evaluation of prostate MRI according to the PIRADS system, thus the clinically performed MRIs did not facilitate the identification of the DIL. The resulting heterogeneity in treatment delivery limits definitive interpretation of efficacy. Second, staging with acetate PET − which is no longer considered standard practice − may have resulted in understaging of nodal disease, although the modality has relatively high specificity for PET-positive lymph nodes [30]. While elective pelvic fields would have covered most microscopic nodal disease, staging limitations remain an important factor when interpreting oncological outcomes. Third, systemic therapy was inferior to modern standards, and absence of androgen receptor signalling inhibitors likely disadvantaged the cohort relative to contemporary trials enrolling similarly high-risk patients. Fourth, the single-arm design precludes direct comparison with standard-dose prostate only radiotherapy or contemporary WPRT protocols. An ongoing case-control study based on the PARAPLY cohort, in which patients are compared with matched controls, together with a larger prospective trial (ISRCTN80146950), will provide additional insight into the comparative effectiveness of the treatment strategy. Finally, the sample size was modest, and the number of events limited, particularly within the very high-risk subgroups. As a result, the precision of the estimates is reduced, and the generalisability of these findings should be interpreted with caution.

## 5 Conclusions

Whole-pelvis radiotherapy with dose escalation to MRI-defined intraprostatic lesions and PET-positive pelvic lymph nodes was well tolerated. The high proportion of patients who did not receive an intraprostatic boost highlights methodological challenges and emphasises the need for accurate imaging interpretation and standardised target-delineation guidelines.

**Funding**

This research was funded by the Cancer Research Foundation in Northern Sweden.

## CRediT authorship contribution statement

**Camilla Thellenberg Karlsson:** Writing – review & editing, Writing – original draft, Supervision, Resources, Project administration, Methodology, Investigation, Funding acquisition, Formal analysis, Conceptualization. **Kristina Notstam:** Writing – review & editing, Writing – original draft, Formal analysis, Data curation. **Björn Tavelin:** Writing – review & editing, Validation, Formal analysis, Data curation. **Kristina Lundqvist:** Writing – review & editing, Formal analysis, Data curation. **Per Fransson:** Writing – review & editing, Methodology, Formal analysis. **Karin Söderkvist:** Writing – review & editing, Writing – original draft, Validation, Supervision, Methodology, Investigation, Data curation, Conceptualization.

## Declaration of competing interest

The authors declare that they have no known competing financial interests or personal relationships that could have appeared to influence the work reported in this paper.
