# Supplementary material for: Outcomes of pelvic radiotherapy with boost strategies in high nodal-risk prostate cancer: A phase 2 prospective trial
Source: Clin Transl Radiat Oncol. 2026 Apr 23;59:101175. doi: 10.1016/j.ctro.2026.101175 (PMC13137043; doi:10.1016/j.ctro.2026.101175)
Supplement: Supplementary Data 1 [file mmc1.pdf]

PARAPLY-studien

PAtients receiving **RA**diotherapy for **P**rostatic cancer, with high risk for **LY**mph node metastasis

2013-03-28 Godkänt EPN v1.0

2013-09-12 (för kännedom till EPN)

2015-02-05 v2.0

Dose escalation with SIB to intraprostatic/lymphatic GTV in high risk prostate cancer

Method

Study design

The present study will be performed as a single institute prospective consecutive phase II clinical trial with matched historical controls of dose escalation to intra prostatic tumor and, if eligible, to radiological lymph node metastasis in the pelvis.
Patients are recruited consecutively during their consultation at Cancer Centre, Umeå University Hospital, after given both oral and written patient information and due time for consideration.
All patients included in the present study will receive external radiotherapy (RT) of 2,2 Gy to 77 Gy to the prostate in 35 fractions and pelvic nodal radiotherapy of 1,6 Gy to 56 Gy in 35 fractions. A simultaneous integrated boost (SIB) of 84 Gy in 35 fractions (fractional dose 2,4 Gy) will in addition be delivered to macroscopic tumor in the prostate. In case of radiological suspected metastasis in one or more of the pelvic lymphatic nodes according to PET/CT-scan an additional SIB of 70 Gy in 35 fractions (fractional dose 2 Gy) will be delivered to PET-positive nodules.
At baseline QoL questionnaire and baseline symptoms will be collected after given informed consent. Renewed QoL and symptom scoring will be collected at end of treatment and during follow up. A historical control group of 100 consecutive patients receiving external radiotherapy to prostate/pelvic lymph nodes without dose escalation to the intra prostatic macroscopic tumor will be used to compare PFS, OS, LC and QoL. Matching criteria is T-stadium, pre-treatment PSA and GS.

Trial period

Patient recruitment is calculated to take 18 months and follow-up time 60 months

Concomitant therapy

Patients will receive hormonal treatment consisting of neo adjuvant LHRH-analog 3 months before start of treatment and 3 months concomitant with radiotherapy. This will be followed by 6 months of anti androgen treatment. In case of suspected lymph node metastasis, individual duration of adjuvant hormonal treatment according to treating physician is allowed.

Patients

86 consecutive patients with high risk prostate cancer at Cancer Centre, Umeå University Hospital planned for external radiotherapy with curative intent that meets the following inclusion criteria and none of the exclusion criteria:

Inclusion:

- Histologically confirmed high risk prostate cancer with a risk of lymphatic spread >15% according to the MSKCC nomogram (1), previously untreated http://nomograms.mskcc.org/Prostate/PreTreatment.aspx
- Written informed consent
- > 18 years
- Fiducial gold markers implanted in the prostate (min 3)

Exclusion:

- Non MR-safe implants or other contraindication to MRI
- WHO PS>1
- Previous pelvic irradiation
- TURP within 6 months
- IPSS >19
- Metastatic disease in skeleton, parenchymal organs or lymph nodes outside the pelvis
- Kreatinin clearance < 30ml/min according to http://www.fass.se/LIF/produktfakta/kreatinin.jsp

Radiation treatment

Radiotherapy

The RT will be delivered with treatment position and fixation according to standard practice of the department. Treatment technique used will be volumetric modulated arc therapy (VMAT) to utilise the benefits of intensity modulated radiation therapy (IMRT) in dose distribution (2-6) and the benefits of VMAT in shortend treatment time (7)
The time limit specified for this protocol from set up portal imaging until end of treatment will be <7 minutes. A review of our previous in house treatment times, from portal imaging until beam off, in our prostate/pelvic nodal VMAT treatments have been performed prior to this study. In 80% of the treatment fractions the treatment time was equal to or less than 7 min. In light of this we consider a margin of 2 mm sufficient for the intra fractional movements of the prostate (7).
A daily online position verification protocol with implanted fiducial markers will be used to minimize positioning errors (8).
The target volume of pelvic lymph nodes is based on the vascular structures of the pelvis according to the RTOG consensus (9). The standard deviations at our treatment centre for prostate/pelvic nodal VMAT treatments with the same pelvic target definitions (Sigma) regarding fiducial markers vs bony structures is 0,5 mm, 1,8 mm and 1,5 mm respectively for lateral, longitudinal and vertical shifts whereas the mean random error (sigma) is 1,2 mm, 2,6 mm and 2,4 mm respectively for lateral, longitudinal and vertical shifts (outliers subtracted from analysis). This will require an additional margin of 7 mm for the PTV-PNV as we position only on the intra prostatic fiducial markers.
At minimum, once a week a cone beam CT on the treatment machine will be performed in order to assess the relation between skeletal/vascular structures and prostate compared to the CT used for dose planning to identity outliers. The action level for treating

physician/investigator to evaluate if a correction of the pelvic radiation volume, e.g. increase the margin, or extra imaging is necessary is a 7 mm deviation from set up exam.
Separate instructions (oral and written) are given to the patients in order to minimise bowel movements and variations in rectal and bladder filling at the visit before the start of RT.

Patient data acquisition

For all patients, a CT based simulation with 2 mm slice thickness shall be made after implantation of fiducial markers in treatment position. In patients with rectum > 4 cm due to faeces or gas, new planning CT and MRI should be performed prior to treatment planning. The bladder should be comfortably filled.
CT images will be obtained from the lower lumbal spine to 10 mm below the tuberositas ischii.
Functional imaging
In case of inclusion in the full protocol, patients will be referred to an 11C-acetate PET/MRI in treatment position before onset of neo adjuvant hormonal treatment. Three additional 11C-acetate PET/MRIwill be performed; after implantation of fiducial markers, in mid-treatment and six months after end of RT. For patients not included in the parallel prognostic imaging protocol, MRI before hormonal treatment and after implantation of the fiducial markers will be performed for target definition. The slice thickness in the planning treatment series is 2 mm.
For all patients, anatomical series consisting of T2 weighted spin-echo based sequences, a T1 weighted gradient echo sequence with DIXON type fat/water separation and a MR angiography. The functional MRI scans include a diffusion weighted EPI sequence (DWI) and a dynamic contrast enhanced spoiled gradient echo sequence (DCE). The DWI sequence is run using two multiple b-values and is reconstructed to an apparent diffusion coefficient (ADC) map that is used for target delineation. The DCE images are analysed using kinetic models and reconstructed to the parameter images used for target definition. The angiography images are registered to the CT image using a rigid mutual information algorithm. All other MRI series are registered to the CT image set using a landmark registration algorithm, with landmarks defined by the implanted gold fiducial markers visible in the T1 weighted MR image and CT. The anatomical series will be used for target delineation in combination with the primary PET scan and planning CT. The primary DWI, will be used in combination with the PET scans to define a macroscopic intra prostatic tumor volume.

VOI-definition

The definition of volumes follows the recommendations made by ICRU in Report 50, 62 and 83 for photon beam therapy (10, 11). Naming of VOI according to national standard adopted from ICRU by SSM 2014:25.

Target volumes

Gross tumor volume intra prostatic, GTVT:
Outlined on the DWI combined with information from the other MRI series, the primary PET scan and histological report, clinical judgment of treating physician with consultant of investigator/radiologist if needed will be used.
Gross tumor volume metastatic lymph node, GTVN1,2..:
Any lymph nodes suspected for macroscopic tumor growth in the primary PET/MRI are to be outlined on the planning CT.
Clinical target volume prostate, CTVT:

The entire prostate including proximal seminal vesicles outlined in CT (with anatomical MR support). Any areas with uncertainties regarding extra capsular growth or tumor invasion in vesicles are to be included.

Clinical target volume vesicula seminalis, CTVT2:

The seminal vesicles outlined on CT (with anatomical MR support) without margin.

Clinical target volume pelvic lymph nodes, CTVN:

The vessels, arterial and vein, will be outlined on CT (with angio sequence MR support) and include bilaterally with a margin of 7 mm in all directions (adjusted for bowel, bladder, bone and muscle): distal common iliac, presacral (S1-S3), external- and internal iliac and the obturator vascular structures. Bony landmarks are L5/S1 interspace (cranial) and superior aspect of the pubic bone (caudal). If applicable, GTVN shall be included in CTVN.

Planning target volume intra prostatic tumor, PTVT_84

An isotrop margin of 2 mm will be added around GTV adjusted so that no part of PTVT_84 extends into urethra, bladder or rectum/anal canal.

Planning target volume protate, PTVT_77

Consists of CTVT and an isotrop margin of 6 mm.

Planning target volume metastatic lymph node, PTVN1,2.._70_:

GTVN_1,2.. with a margin of 7 mm in all directions if applicable.

Planning target volume pelvic lymph node, vesicula seminalis, PTV_56

Consists of CTVT2 and CTVN with a margin of 7 mm in all directions.

Note!

If automatic margin expansion is used, check the margin in the
cranio-caudal (cc) direction.
Delineation of treatment volumes may be performed by treating physician, however it is recommended that treatment plan is approved by investigator before start of treatment.

Organ at risk

Rectum and AnalCanal

The rectal volume shall be outlined as the outer contour of the rectum, i.e. including the rectal wall. The extension of the segmentation shall, if applicable, be 5 cm in the cranial direction from the centre of the prostate and in the caudal direction down to and including the anus. , adjusted to 10 cm cranially-caudally. The anal canal shall be outlined as a separate OR consisting of the most caudal 4 cm of the rectum.

BowelBag

The potential space for the bowel should be outlined as possible OAR. Borders are the abdominal wall anteriorly, pelvic sidewalls laterally (excluding the pelvic lymph nodes), superiorly to one slice above the last image where the lymph nodes are outlined and the inferior margins goes to the top of the CTV-P on the side of the bladder for the part of the bowel that may fall down there.

Bladder

The entire bladder from neck to dome, including the bladder wall

Urethra

Outlined as a tube with a diameter of 3mm, cranially from the neck of the bladder stretching the whole length of the PTV-P caudally, it is recommended to approximate the urethra in sagital series initially.

FemoralHead_L/R

Without margin

PenileBulb

The penile bulb shall be outlined on the CT images (MR guidance is recommended).

Dose specifications

Prescribed average dose to CTVT: 2,2 Gy to 77 Gy, 5 fractions per week with a total of 35 fractions.
Prescribed average dose to CTV2 and CTVN: 1,6 Gy to 56 Gy in 35 fractions.
Prescribed average dose to GTV: 2,4 Gy to 84 Gy in 35 fractions.
Prescribed average dose to GTVN1,2..: 2Gy to 70 Gy in 35 fractions.

Dose-volume objectives

If not otherwise specified, prescribed dose refers to CTVT.

Priority	VOI	D-V objective	
1	CTVT	$D_{98\%} \geq 74.7$ Gy	The "near minimum dose" to CTVT shall be greater than or equal to 97% of the prescribed dose.
2	PTVT_77	$V_{73Gy} \geq 95\%$	The 95% isodose shall cover at least 95% of PTV_77.
3	Rectum	$V_{69Gy} \leq 15\%$	Less than 15 % of the outlined rectal volume should receive doses greater than 90% of the prescribed dose.
4	PTVT_77	$D_{98\%} \geq 70$ Gy	The "near minimum dose" to PTV_77 should be greater than or equal to 95% of the prescribed dose.
5	Rectum	$V_{58Gy} \leq 35\%$	Less than 35 % of the outlined rectal volume should receive doses greater than 75% of the prescribed dose.
6	FemoralHead	$D_{2\%} \leq 54$ Gy	The "near maximum dose" to the femoral heads should be less than or equal to 70% of the prescribed dose.
7	BowelBag	$V_{45Gy} \leq 150$cc<br>$V_{30Gy} \leq 300$cc<br>$D_{2\%} \leq 53.2$ Gy	The "near maximum dose" to the bowel bag should be no more than 95% of the prescribed dose CTVT and N.
8	Urethra	$D_{2\%} \leq 77$ Gy	The "near maximum dose" to the urethra should be less than or equal to the prescribed dose.
9	PTV_56	$V_{53Gy} \geq 95\%$	The 95% isodose should cover at least 95% of CTVT and N.
11	Body	$D_{2\%} \leq 81.9\%$	The global "near maximum dose" *outside of PTVT_84* should be less than or equal to 105% of the prescribed dose.

12	Bladder	$D_{mean} \leq 61,5$	Mean dose to bladder should be less or equal to 80 % of prescribed dose.

Endpoints

Primary endpoint

Progression Free Survival
Progression free survival is defined as the length of time from inclusion to the first documentation of one of the following:

PSA progression defined according to American Society for Therapeutic Radiology and Oncology (ASTRO) Phoenix definition: nadir PSA + 2 ng/mL (on three consecutive measurements with at least one month between each)
or
Death due to any cause
or
Radiological disease progression, locally or as metastatic disease.

Due to potential "PSA bounce" during the first 24 months after treatment, a PSA rise >2 ng/mL may not be deemed as progression by the treating physician until after the clinical window of a bounce is excluded. The date for progression will then be corrected.

Local Control
Local control is defined as negative biopsy 36 months after end of radiotherapy.

Secondary endpoint

Overall Survival
Overall survival is defined as the length of time from randomization to death from any cause.

Safety

At every follow up timepoint all adverse events will be documented and every effort will be made to collect patient charts if the patient has been treated outside our on centre.

Quality of Life

Follow up

During the first year after treatment the patients will be seen by treating oncologist for clinical evaluation every 6 months (± 14 days). Additional visits at treating physician's discretion. During the follow up between 12-60 months the patient may be referred to their home clinic and followed by urologist every 6 months (± 28 days) or until progression. The 36 months-visit however, will take place at NUS, where a biopsy to investigate local control will be performed.

Quality of Life

Patients will receive QoL forms before start of hormonal treatment, at end of radiotherapy and at follow-up time points 6 months, 1, 2, 3 year and 5 years. PCSS form, a validated form of symptom evaluation will be used

Withdrawal from treatment

The patient can at any time withdraw from treatment. The date and reason for withdrawal should be reported in the CRF. If a patient is lost to follow up the date and reason for this should be reported in the CRF. Loss of follow-up does not mean that the patient leaves the study. All patients that are randomised should be followed until the end points have been reached - irrespective of further treatment regimes and in accordance with the intention of treatment strategy.

Flow Chart

See App 1

Side effects

Side effects should be reported according to GCP and noted in the CRF. Symptoms related to proven tumour progression should not be considered as side effects. Grading will be done according to RTOG radiotherapy scoring system. For events except bladder and small/large intestinal we will use Common Terminology Criteria for Adverse Events (CTC-AE) version 4.1.

Adverse Events (AE)

Reported in CRF.

Serious Adverse Events (SAE)

Serious side effects during the study should be reported to Klinisk Forskningsenhet, KFE, Norrlands universitetssjukhus. Hospitalisation due to suspected **serious side effects related to treatment** should be reported within 10 days to Klinisk Forskningsenhet, by FAX. Symptoms and death related to proven tumour progression should not be considered as serious side effects. Serious side effects are those that are life-threatening, or may give serious permanent damage or dysfunction of a magnitude that need hospital care. Grading will be done according to RTOG/EORTC. For events except bladder and small/large intestinal use Common Terminology Criteria for Adverse Events (CTC-AE) version 4.1.

Data management and clinical procedures

Case Record Form (CRF)

A paper version will be used.

Data management

KFE

Statistical procedures

Power considerations

In this extremely high risk population there is a considerable risk of relapse. Calculating on an improvement of 20% with dose-escalation and irradiation of lymph-nodes with boost to radiologically pathologic nodes we would need 86 patients to detect an increased freedom from relapse at three years with 80 % power and a two-sided significance level of 0,05.

Statistical methods

Prospective phase II clinical trial with matched historical controls.

Report and communication of results

Publication

All presentations of data from the study, in the form of lectures or publications, should only be made after agreement with the study-coordinators. The results of the study will be submitted to an internationally recognized medical journal. It is the intention of the present study that analysis of the primary endpoint should not be made until the required number of events has been obtained. If solid statistical data emerge, or other exceptional circumstances occur they may, however, be published earlier after approval by the study-coordinators.

Refences

1. Cagiannos I, Karakiewicz P, Eastham JA, Ohori M, Rabbani F, Gerigk C, et al. A preoperative nomogram identifying decreased risk of positive pelvic lymph nodes in patients with prostate cancer. J Urol. 2003;170(5):1798-803. Epub 2003/10/09.
2. Myrehaug S, Chan G, Craig T, Weinberg V, Cheng C, Roach M, 3rd, et al. A treatment planning and acute toxicity comparison of two pelvic nodal volume delineation techniques and delivery comparison of intensity-modulated radiotherapy versus volumetric modulated arc therapy for hypofractionated high-risk prostate cancer radiotherapy. Int J Radiat Oncol Biol Phys. 2012;82(4):e657-62. Epub 2012/01/17.
3. Chung HT, Xia P, Chan LW, Park-Somers E, Roach M, 3rd. Does image-guided radiotherapy improve toxicity profile in whole pelvic-treated high-risk prostate cancer? Comparison between IG-IMRT and IMRT. Int J Radiat Oncol Biol Phys. 2009;73(1):53-60. Epub 2008/05/27.
4. Wang-Chesebro A, Xia P, Coleman J, Akazawa C, Roach M, 3rd. Intensity-modulated radiotherapy improves lymph node coverage and dose to critical structures compared with three-dimensional conformal radiation therapy in clinically localized prostate cancer. Int J Radiat Oncol Biol Phys. 2006;66(3):654-62. Epub 2006/10/03.
5. Luxton G, Hancock SL, Boyer AL. Dosimetry and radiobiologic model comparison of IMRT and 3D conformal radiotherapy in treatment of carcinoma of the prostate. Int J Radiat Oncol Biol Phys. 2004;59(1):267-84. Epub 2004/04/20.
6. Pinkawa M, Piroth MD, Holy R, Djukic V, Klotz J, Krenkel B, et al. Combination of dose escalation with technological advances (intensity-modulated and image-guided radiotherapy) is not associated with increased morbidity for patients with prostate cancer. Strahlenther Onkol. 2011;187(8):479-84. Epub 2011/07/27.
7. Kotte AN, Hofman P, Lagendijk JJ, van Vulpen M, van der Heide UA. Intrafraction motion of the prostate during external-beam radiation therapy: analysis of 427 patients with implanted fiducial markers. Int J Radiat Oncol Biol Phys. 2007;69(2):419-25. Epub 2007/05/22.
8. van der Heide UA, Kotte AN, Dehnad H, Hofman P, Lagenijk JJ, van Vulpen M. Analysis of fiducial marker-based position verification in the external beam radiotherapy of patients with prostate cancer. Radiother Oncol. 2007;82(1):38-45. Epub 2006/12/05.
9. Lawton CA, Michalski J, El-Naqa I, Buyyounouski MK, Lee WR, Menard C, et al. RTOG GU Radiation oncology specialists reach consensus on pelvic lymph node volumes for high-risk prostate cancer. Int J Radiat Oncol Biol Phys. 2009;74(2):383-7. Epub 2008/10/25.
10. International Commission on Radiation Units and Measurements. Prescribing, recording and reporting photon beam therapy report 50. Bethesda MD; 1993.
11. International Commission on Radiation Units and Measurements. Prescribing, recording, and reporting photon beam therapy (Supplement to ICRU Report 50). Bethesda MD; 1999.

Flowchart

**Investigation**	**3 months Pre RT**	**RT start**	**Mid RT**	**End of RT**	**6 months after end of RT**	**12 months after end of RT**	**18 months after end of RT**	**24 months after end of RT**	**30 months after end of RT**	**36 months after end of RT**	**Every 6 month until endpoint**
Inclusion/Exclusion	X										
Informed consent	X										
Androgen deprivation therapy GnRH	X	X	X	X	End						
Androgen deprivation therapy, antiandrogen				Start		End Continuous optional, dr choice					
PET-MRI (imaging intense)	X	X	X		X					X	
MRI (imaging non intense)	X	X									
Blood tests: PSA, Hb, Creatinine, Testosterone, ALP	X	X			X	X	X	X	X	X	
UCAN-samples	X	X		X	X		X			X	
Quality of Life	X			X	X	X		X		X	5 years*
Physician, safety	X	X		X	X	X	X	X	X	X	
Instructions on bowel, bladder filling. Oral and written		X									
Prostate Biopsy, optional		X								X	
