# Supplementary material for: Outcomes of pelvic radiotherapy with boost strategies in high nodal-risk prostate cancer: A phase 2 prospective trial
Source: Clin Transl Radiat Oncol. 2026 Apr 23;59:101175. doi: 10.1016/j.ctro.2026.101175 (PMC13137043; doi:10.1016/j.ctro.2026.101175)
Supplement: Supplementary Data 2 [file mmc2.docx]

**Table S1** **Dosimetric parameters for the GTV**

This table summarises dose-volume parameters for the dominant intraprostatic lesion (GTV) among the 31 patients with available dosimetric data, including GTV volume and the Dmin, Dmax, and Dmean doses.

| NR | GTV_Volume(cm3) | GTV_DMax (Gy) | GTV_DMin (Gy) | GTV_DMean (Gy) |
| --- | --- | --- | --- | --- |
| 1 | 2,5 | 84,0 | 80,5 | 83,1 |
| 2 | 3,9 | 85,1 | 80,7 | 83,5 |
| 3 | 2,4 | 85,7 | 76,9 | 82,7 |
| 5 | 17,5 | 85,9 | 75,3 | 83,2 |
| 6 | 3,9 | 85,9 | 74,3 | 82,9 |
| 9 | 3,2 | 85,9 | 75,4 | 82,9 |
| 12 | 7,6 | 85,7 | 73,6 | 81,5 |
| 16 | 3,4 | 85,8 | 78,1 | 83,6 |
| 19 | 3,9 | 85,7 | 74,7 | 83,3 |
| 20 | 0,4 | 86,3 | 81,1 | 84,7 |
| 27 | 0,3 | 84,3 | 79,6 | 83,1 |
| 34 | 0,3 | 85,1 | 82,7 | 84,1 |
| 38 | 1,3 | 85,6 | 78,6 | 84,0 |
| 43 | 1,7 | 86,4 | 78,6 | 84,0 |
| 45 | 2,8 | 85,6 | 76,3 | 82,8 |
| 46 | 1,2 | 85,2 | 76,3 | 82,4 |
| 49 | 1,5 | 85,6 | 77,3 | 83,7 |
| 51 | 1,6 | 85,1 | 77,3 | 83,3 |
| 52 | 3,5 | 85,5 | 77,0 | 83,4 |
| 54 | 0,8 | 85,0 | 78,2 | 83,2 |
| 63 | 1,1 | 84,8 | 81,4 | 83,6 |
| 66 | 1,9 | 85,9 | 75,7 | 81,9 |
| 67 | 3,2 | 86,1 | 75,8 | 83,6 |
| 68 | 0,7 | 85,0 | 81,3 | 83,8 |
| 69 | 0,7 | 85,1 | 77,0 | 82,2 |
| 70 | 3,9 | 85,5 | 80,5 | 84,1 |
| 71 | 2,6 | 85,4 | 75,1 | 82,2 |
| 75 | 2,6 | 85,2 | 79,1 | 83,6 |
| 76 | 0,4 | 85,5 | 80,3 | 84,1 |
| 78 | 2,5 | 86,0 | 73,2 | 82,4 |
| 83 | 10,7 | 85,4 | 77,3 | 83,2 |
