# Supplementary material for: Outcomes of pelvic radiotherapy with boost strategies in high nodal-risk prostate cancer: A phase 2 prospective trial
Source: Clin Transl Radiat Oncol. 2026 Apr 23;59:101175. doi: 10.1016/j.ctro.2026.101175 (PMC13137043; doi:10.1016/j.ctro.2026.101175)
Supplement: Supplementary Data 3 [file mmc3.docx]

**Table S2. Comparison of clinician‑reported toxicity and patient‑reported outcomes between patients treated with and without an intraprostatic boost**

| **Time_DE * Grade_GU Crosstabulation** | | | | | | | | |
| --- | --- | --- | --- | --- | --- | --- | --- | --- |
| Count |  |  |  |  |  |  |  |  |
| Prostate-boost | | | Grade_GU | | | | | Total |
|  |  |  | 0 | 1 | 2 | 3 | 4 |  |
| Non-boost | time_DE | 0 | 27 | 6 | 2 | 0 | 0 | 35 |
|  |  | 1 | 7 | 17 | 9 | 1 | 2 | 36 |
|  |  | 6 | 20 | 10 | 4 | 2 | 0 | 36 |
|  |  | 12 | 23 | 7 | 1 | 2 | 0 | 33 |
|  |  | 18 | 26 | 4 | 2 | 1 | 0 | 33 |
|  |  | 24 | 26 | 2 | 3 | 2 | 0 | 33 |
|  |  | 30 | 23 | 3 | 2 | 1 | 0 | 29 |
|  |  | 36 | 25 | 3 | 2 | 0 | 0 | 30 |
|  | Total | | 177 | 52 | 25 | 9 | 2 | 265 |
| Boost | time_DE | 0 | 33 | 5 | 2 | 0 |  | 40 |
|  |  | 1 | 8 | 20 | 10 | 4 |  | 42 |
|  |  | 6 | 31 | 8 | 3 | 0 |  | 42 |
|  |  | 12 | 33 | 4 | 3 | 0 |  | 40 |
|  |  | 18 | 29 | 6 | 2 | 0 |  | 37 |
|  |  | 24 | 30 | 7 | 3 | 0 |  | 40 |
|  |  | 30 | 32 | 4 | 3 | 0 |  | 39 |
|  |  | 36 | 28 | 5 | 6 | 0 |  | 39 |
|  | Total | | 224 | 59 | 32 | 4 |  | 319 |
| **Ranks** | | | | | |  |  |  |
| time_months_DE | | Prostate-boost | N | Mean Rank | Sum of Ranks |  |  |  |
| 0 | Grade_GU | Without | 35 | 39,04 | 1366,50 |  |  |  |
|  |  | With | 40 | 37,09 | 1483,50 |  |  |  |
|  |  | Total | 75 |  |  |  |  |  |
| 1 | Grade_GU | Without | 36 | 39,47 | 1421,00 |  |  |  |
|  |  | With | 42 | 39,52 | 1660,00 |  |  |  |
|  |  | Total | 78 |  |  |  |  |  |
| 6 | Grade_GU | Without | 36 | 43,67 | 1572,00 |  |  |  |
|  |  | With | 42 | 35,93 | 1509,00 |  |  |  |
|  |  | Total | 78 |  |  |  |  |  |
| 12 | Grade_ GU | Without | 33 | 39,52 | 1304,00 |  |  |  |
|  |  | With | 40 | 34,93 | 1397,00 |  |  |  |
|  |  | Total | 73 |  |  |  |  |  |
| 18 | Grade_ GU | Without | 33 | 35,61 | 1175,00 |  |  |  |
|  |  | With | 37 | 35,41 | 1310,00 |  |  |  |
|  |  | Total | 70 |  |  |  |  |  |
| 24 | Grade_ GU | Without | 33 | 36,77 | 1213,50 |  |  |  |
|  |  | With | 40 | 37,19 | 1487,50 |  |  |  |
|  |  | Total | 73 |  |  |  |  |  |
| 30 | Grade_ GU | Without | 29 | 35,14 | 1019,00 |  |  |  |
|  |  | With | 39 | 34,03 | 1327,00 |  |  |  |
|  |  | Total | 68 |  |  |  |  |  |
| 36 | Grade_ GU | Without | 30 | 32,62 | 978,50 |  |  |  |
|  |  | With | 39 | 36,83 | 1436,50 |  |  |  |
|  |  | Total | 69 |  |  |  |  |  |

| **Test Statistics^a^** | | |
| --- | --- | --- |
| Time_months_DE | | Grade_ GU |
| 0 | Mann-Whitney U | 663,500 |
|  | Wilcoxon W | 1483,500 |
|  | Z | -0,557 |
|  | Asymp. Sig. (2-tailed) | 0,578 |
| 1 | Mann-Whitney U | 755,000 |
|  | Wilcoxon W | 1421,000 |
|  | Z | -0,011 |
|  | Asymp. Sig. (2-tailed) | 0,991 |
| 6 | Mann-Whitney U | 606,000 |
|  | Wilcoxon W | 1509,000 |
|  | Z | -1,787 |
|  | Asymp. Sig. (2-tailed) | 0,074 |
| 12 | Mann-Whitney U | 577,000 |
|  | Wilcoxon W | 1397,000 |
|  | Z | -1,246 |
|  | Asymp. Sig. (2-tailed) | 0,213 |
| 18 | Mann-Whitney U | 607,000 |
|  | Wilcoxon W | 1310,000 |
|  | Z | -0,058 |
|  | Asymp. Sig. (2-tailed) | 0,954 |
| 24 | Mann-Whitney U | 652,500 |
|  | Wilcoxon W | 1213,500 |
|  | Z | -0,112 |
|  | Asymp. Sig. (2-tailed) | 0,910 |
| 30 | Mann-Whitney U | 547,000 |
|  | Wilcoxon W | 1327,000 |
|  | Z | -0,335 |
|  | Asymp. Sig. (2-tailed) | 0,738 |
| 36 | Mann-Whitney U | 513,500 |
|  | Wilcoxon W | 978,500 |
|  | Z | -1,174 |
|  | Asymp. Sig. (2-tailed) | 0,241 |
| a. Grouping Variable: Prostate-boost | | |

**Comparison of gastrointestinal (GI) toxicity between patients treated with and without an intraprostatic boost**

| **Time_DE * Grade_GI Crosstabulation** | | | | | | | |
| --- | --- | --- | --- | --- | --- | --- | --- |
| Count |  |  |  |  |  |  |  |
| Prostate-boost | | | Grade_GI | | | | Total |
|  |  |  | 0 | 1 | 2 | 3 |  |
| Non-boost | time_DE | 0 | 33 | 0 | 0 | 0 | 33 |
|  |  | 1 | 12 | 19 | 5 | 0 | 36 |
|  |  | 6 | 27 | 5 | 3 | 1 | 36 |
|  |  | 12 | 21 | 9 | 2 | 0 | 32 |
|  |  | 18 | 24 | 9 | 0 | 0 | 33 |
|  |  | 24 | 23 | 10 | 0 | 0 | 33 |
|  |  | 30 | 18 | 8 | 2 | 0 | 28 |
|  |  | 36 | 22 | 8 | 1 | 0 | 31 |
|  | Total | | 180 | 68 | 13 | 1 | 262 |
| Boost | time_DE | 0 | 35 | 1 | 0 | 0 | 36 |
|  |  | 1 | 11 | 18 | 12 | 1 | 42 |
|  |  | 6 | 27 | 15 | 0 | 0 | 42 |
|  |  | 12 | 34 | 5 | 1 | 0 | 40 |
|  |  | 18 | 29 | 7 | 1 | 0 | 37 |
|  |  | 24 | 33 | 7 | 0 | 0 | 40 |
|  |  | 30 | 31 | 6 | 1 | 0 | 38 |
|  |  | 36 | 26 | 11 | 1 | 0 | 38 |
|  | Total | | 226 | 70 | 16 | 1 | 313 |

| **Ranks** | | | | | |
| --- | --- | --- | --- | --- | --- |
| time_months_DE | | Prostate-boost | N | Mean Rank | Sum of Ranks |
| 0 | Grade_GI | Without | 33 | 34,50 | 1138,50 |
|  |  | With | 36 | 35,46 | 1276,50 |
|  |  | Total | 69 |  |  |
| 1 | Grade_ GI | Without | 36 | 35,75 | 1287,00 |
|  |  | With | 42 | 42,71 | 1794,00 |
|  |  | Total | 78 |  |  |
| 6 | Grade_ GI | Without | 36 | 38,08 | 1371,00 |
|  |  | With | 42 | 40,71 | 1710,00 |
|  |  | Total | 78 |  |  |
| 12 | Grade_ GI | Without | 32 | 40,39 | 1292,50 |
|  |  | With | 40 | 33,39 | 1335,50 |
|  |  | Total | 72 |  |  |
| 18 | Grade_ GI | Without | 33 | 36,41 | 1201,50 |
|  |  | With | 37 | 34,69 | 1283,50 |
|  |  | Total | 70 |  |  |
| 24 | Grade_ GI | Without | 33 | 39,56 | 1305,50 |
|  |  | With | 40 | 34,89 | 1395,50 |
|  |  | Total | 73 |  |  |
| 30 | Grade_ GI | Without | 28 | 36,86 | 1032,00 |
|  |  | With | 38 | 31,03 | 1179,00 |
|  |  | Total | 66 |  |  |
| 36 | Grade_ GI | Without | 31 | 34,56 | 1071,50 |
|  |  | With | 38 | 35,36 | 1343,50 |
|  |  | Total | 69 |  |  |

| **Test Statistics^a^** | | |
| --- | --- | --- |
| time_months_DE | | Grade_GI |
| 0 | Mann-Whitney U | 577,500 |
|  | Wilcoxon W | 1138,500 |
|  | Z | -0,957 |
|  | Asymp. Sig. (2-tailed) | 0,338 |
| 1 | Mann-Whitney U | 621,000 |
|  | Wilcoxon W | 1287,000 |
|  | Z | -1,461 |
|  | Asymp. Sig. (2-tailed) | 0,144 |
| 6 | Mann-Whitney U | 705,000 |
|  | Wilcoxon W | 1371,000 |
|  | Z | -0,633 |
|  | Asymp. Sig. (2-tailed) | 0,526 |
| 12 | Mann-Whitney U | 515,500 |
|  | Wilcoxon W | 1335,500 |
|  | Z | -1,908 |
|  | Asymp. Sig. (2-tailed) | 0,056 |
| 18 | Mann-Whitney U | 580,500 |
|  | Wilcoxon W | 1283,500 |
|  | Z | -0,474 |
|  | Asymp. Sig. (2-tailed) | 0,635 |
| 24 | Mann-Whitney U | 575,500 |
|  | Wilcoxon W | 1395,500 |
|  | Z | -1,279 |
|  | Asymp. Sig. (2-tailed) | 0,201 |
| 30 | Mann-Whitney U | 438,000 |
|  | Wilcoxon W | 1179,000 |
|  | Z | -1,600 |
|  | Asymp. Sig. (2-tailed) | 0,110 |
| 36 | Mann-Whitney U | 575,500 |
|  | Wilcoxon W | 1071,500 |
|  | Z | -0,203 |
|  | Asymp. Sig. (2-tailed) | 0,839 |
| a. Grouping Variable: Prostate-boost | | |

**Comparison of patient‑reported urinary problems between patients treated with and without an intraprostatic boost**

| **Report** | | | | |
| --- | --- | --- | --- | --- |
| PRO_urinary |  |  |  |  |
| Time point | Prostate-boost | N | Mean | Std. Error of Mean |
| Baseline | Without | 29 | 2,31 | 0,474 |
|  | With | 35 | 2,00 | 0,391 |
|  | Total | 64 | 2,14 | 0,301 |
| End of RT | Without | 32 | 4,84 | 0,492 |
|  | With | 30 | 4,33 | 0,598 |
|  | Total | 62 | 4,60 | 0,383 |
| 6 m | Without | 28 | 3,32 | 0,561 |
|  | With | 34 | 4,00 | 0,524 |
|  | Total | 62 | 3,69 | 0,382 |
| 12 m | Without | 31 | 1,97 | 0,389 |
|  | With | 29 | 2,00 | 0,441 |
|  | Total | 60 | 1,98 | 0,291 |
| 24 m | Without | 29 | 2,10 | 0,436 |
|  | With | 36 | 1,97 | 0,407 |
|  | Total | 65 | 2,03 | 0,296 |
| 36 m | Without | 28 | 2,00 | 0,463 |
|  | With | 32 | 2,22 | 0,442 |
|  | Total | 60 | 2,12 | 0,317 |
| 60 m | Without | 23 | 3,09 | 0,589 |
|  | With | 30 | 2,13 | 0,527 |
|  | Total | 53 | 2,55 | 0,395 |
| Total | Without | 200 | 2,82 | 0,194 |
|  | With | 226 | 2,65 | 0,189 |
|  | Total | 426 | 2,73 | 0,135 |

| **Test Statistics^a^** | | |
| --- | --- | --- |
| Time point | | PRO_urinary |
| Baseline | Mann-Whitney U | 469,500 |
|  | Wilcoxon W | 1099,500 |
|  | Z | -0,526 |
|  | Asymp. Sig. (2-tailed) | 0,599 |
| End of RT | Mann-Whitney U | 432,500 |
|  | Wilcoxon W | 897,500 |
|  | Z | -0,673 |
|  | Asymp. Sig. (2-tailed) | 0,501 |
| 6 m | Mann-Whitney U | 407,500 |
|  | Wilcoxon W | 813,500 |
|  | Z | -0,979 |
|  | Asymp. Sig. (2-tailed) | 0,328 |
| 12 m | Mann-Whitney U | 444,500 |
|  | Wilcoxon W | 879,500 |
|  | Z | -0,076 |
|  | Asymp. Sig. (2-tailed) | 0,939 |
| 24 m | Mann-Whitney U | 493,000 |
|  | Wilcoxon W | 1159,000 |
|  | Z | -0,398 |
|  | Asymp. Sig. (2-tailed) | 0,690 |
| 36 m | Mann-Whitney U | 420,500 |
|  | Wilcoxon W | 826,500 |
|  | Z | -0,420 |
|  | Asymp. Sig. (2-tailed) | 0,675 |
| 60 m | Mann-Whitney U | 259,000 |
|  | Wilcoxon W | 724,000 |
|  | Z | -1,592 |
|  | Asymp. Sig. (2-tailed) | 0,111 |
| a. Grouping Variable: Prostate-boost | | |

**Comparison of patient‑reported intestinal problems between patients treated with and without an intraprostatic boost**

| **Report** | | | | |
| --- | --- | --- | --- | --- |
| PRO_intestinal |  |  |  |  |
| Time point | Prostate-boost | N | Mean | Std. Error of Mean |
| Baseline | Without | 30 | 1,37 | 0,456 |
|  | With | 38 | 0,29 | 0,192 |
|  | Total | 68 | 0,76 | 0,235 |
| End of RT | Without | 34 | 4,18 | 0,477 |
|  | With | 32 | 4,03 | 0,581 |
|  | Total | 66 | 4,11 | 0,371 |
| 6 m | Without | 31 | 2,87 | 0,560 |
|  | With | 33 | 2,30 | 0,488 |
|  | Total | 64 | 2,58 | 0,369 |
| 12 m | Without | 32 | 2,66 | 0,517 |
|  | With | 29 | 1,45 | 0,367 |
|  | Total | 61 | 2,08 | 0,329 |
| 24 m | Without | 26 | 2,15 | 0,439 |
|  | With | 35 | 1,49 | 0,313 |
|  | Total | 61 | 1,77 | 0,261 |
| 36 m | Without | 28 | 2,14 | 0,479 |
|  | With | 33 | 1,61 | 0,396 |
|  | Total | 61 | 1,85 | 0,306 |
| 60 m | Without | 23 | 1,87 | 0,463 |
|  | With | 28 | 2,07 | 0,488 |
|  | Total | 51 | 1,98 | 0,337 |
| Total | Without | 204 | 2,53 | 0,194 |
|  | With | 228 | 1,85 | 0,169 |
|  | Total | 432 | 2,17 | 0,129 |

| **Test Statistics^a^** | | |
| --- | --- | --- |
| Time point | | PRO_intestinal |
| Baseline | Mann-Whitney U | 432,500 |
|  | Wilcoxon W | 1173,500 |
|  | Z | -2,404 |
|  | Asymp. Sig. (2-tailed) | 0,016 |
| End of RT | Mann-Whitney U | 510,500 |
|  | Wilcoxon W | 1038,500 |
|  | Z | -0,432 |
|  | Asymp. Sig. (2-tailed) | 0,665 |
| 6 m | Mann-Whitney U | 454,500 |
|  | Wilcoxon W | 1015,500 |
|  | Z | -0,794 |
|  | Asymp. Sig. (2-tailed) | 0,427 |
| 12 m | Mann-Whitney U | 352,500 |
|  | Wilcoxon W | 787,500 |
|  | Z | -1,672 |
|  | Asymp. Sig. (2-tailed) | 0,095 |
| 24 m | Mann-Whitney U | 384,500 |
|  | Wilcoxon W | 1014,500 |
|  | Z | -1,066 |
|  | Asymp. Sig. (2-tailed) | 0,286 |
| 36 m | Mann-Whitney U | 410,000 |
|  | Wilcoxon W | 971,000 |
|  | Z | -0,809 |
|  | Asymp. Sig. (2-tailed) | 0,419 |
| 60 m | Mann-Whitney U | 311,500 |
|  | Wilcoxon W | 587,500 |
|  | Z | -0,210 |
|  | Asymp. Sig. (2-tailed) | 0,833 |
| a. Grouping Variable: Prostate-boost | | |
