# Supplementary material for: Outcomes of pelvic radiotherapy with boost strategies in high nodal-risk prostate cancer: A phase 2 prospective trial
Source: Clin Transl Radiat Oncol. 2026 Apr 23;59:101175. doi: 10.1016/j.ctro.2026.101175 (PMC13137043; doi:10.1016/j.ctro.2026.101175)
Supplement: Supplementary Data 4 [file mmc4.docx]

**Table S3. Clinically relevant deterioration**
Proportion of patients with clinically relevant deterioration (≥ 1 from baseline) in urinary and bowel PCSS items at 5 years.

| PCSS | Yes | | | | | | MeanDiff | p-value |
| --- | --- | --- | --- | --- | --- | --- | --- | --- |
| Overall bother from all urinary symptoms | 21 | / | 47 | ( | 45% | ) | 0,766 | 0,082 |
| Urinary frequency per day | 13 | / | 46 | ( | 28% | ) | -0,696 | 0,016 |
| Nocturia (frequncy) | 23 | / | 42 | ( | 55% | ) | 0,714 | 0,001 |
| Pain while urinating | 8 | / | 47 | ( | 17% | ) | 0,085 | 0,710 |
| Starting problems | 11 | / | 48 | ( | 23% | ) | -0,354 | 0,227 |
| Weak stream | 15 | / | 48 | ( | 31% | ) | -0,229 | 0,493 |
| Urinary incontinence | 12 | / | 48 | ( | 25% | ) | 0,333 | 0,169 |
| Stress incontinence | 12 | / | 49 | ( | 24% | ) | 0,306 | 0,058 |
| Urgency | 15 | / | 47 | ( | 32% | ) | -0,213 | 0,578 |
| Rush to toilet to pass urine | 18 | / | 47 | ( | 38% | ) | 0,383 | 0,381 |
| Urge incontinence | 8 | / | 49 | ( | 16% | ) | -0,102 | 0,728 |
| Leakage of stools when passing urine | 8 | / | 49 | ( | 16% | ) | 0,408 | 0,105 |
| Limitation in daily activity caused by urinary symptoms | 15 | / | 49 | ( | 31% | ) | 0,265 | 0,313 |
| Emtying bladder | 16 | / | 49 | ( | 33% | ) | -0,327 | 0,414 |
| Overall bother from all bowel symptoms | 23 | / | 49 | ( | 47% | ) | 1,102 | 0,015 |
| Stool frequency | 20 | / | 49 | ( | 41% | ) | 0,551 | 0,011 |
| Rush to toilet in morning because of bowel movements | 19 | / | 51 | ( | 37% | ) | 0,667 | 0,075 |
| Stool leakage | 16 | / | 51 | ( | 31% | ) | 0,667 | 0,004 |
| Planning of toilet visits | 16 | / | 51 | ( | 31% | ) | 1,000 | 0,009 |
| Flatulence (excessive gas) | 19 | / | 50 | ( | 38% | ) | 0,440 | 0,213 |
| Bowel cramp | 8 | / | 51 | ( | 16% | ) | 0,294 | 0,058 |
| Mucus | 10 | / | 51 | ( | 20% | ) | 0,294 | 0,100 |
| Blood in stools | 13 | / | 51 | ( | 25% | ) | 0,627 | 0,003 |
| Limitation in daily activity caused by bowel symptoms | 16 | / | 50 | ( | 32% | ) | 1,020 | 0,004 |
